# Supplementary material for: Comparison of diversity and composition of macrofungal species between intensive mushroom harvesting and non-harvesting areas in Oaxaca, Mexico
Source: PeerJ. 2019 Dec 18;7:e8325. doi: 10.7717/peerj.8325 (PMC6967021; doi:10.7717/peerj.8325)
Supplement: Supplemental Information 1 — Edible species are marked with * [file peerj-07-8325-s001.docx]

Appendix A. Macromycete species, Families, Orders and Phyla recorded within the studied area at Northwestern Oaxaca, Mexico. “sp., sp. 1, sp. 2, sp. 3…” indicate species unidentified (morphospecies). Edible species are marked with *

| **Species** | **Family** | **Order** | **Phylum** |
| --- | --- | --- | --- |
| *Hypoxylon* sp. | Hypoxylaceae | Xylariales | Ascomycota |
| *Leotia lubrica Fr.* | Leotiaceae | Helotiales | Ascomycota |
| *Otidea alutacea* (Pers.) Massee | Pyronemataceae | Pezizales | Ascomycota |
| *Tolypocladium sp.* | Ophiocordycipitaceae | Hypocreales | Ascomycota |
| Agaricales |  | Agaricales | Basidiomycota |
| *Agrocybe* sp. | Strophariaceae | Agaricales | Basidiomycota |
| *Albatrellus ellisii* (Berk.) Pouzar* | Albatrellaceae | Russulales | Basidiomycota |
| *Albatrellus* sp. | Albatrellaceae | Russulales | Basidiomycota |
| *Amanita* aff. *phalloides* | Amanitaceae | Agaricales | Basidiomycota |
| *Amanita basii* Guzmán & Ram.-Guill.* | Amanitaceae | Agaricales | Basidiomycota |
| *Amanita flavoconia* G.F. Atk. | Amanitaceae | Agaricales | Basidiomycota |
| *Amanita fulva* Fr. | Amanitaceae | Agaricales | Basidiomycota |
| *Amanita* G. *arocheae* | Amanitaceae | Agaricales | Basidiomycota |
| *Amanita* G. *pachycola* | Amanitaceae | Agaricales | Basidiomycota |
| *Amanita gemmata* (Fr.) Bertill. | Amanitaceae | Agaricales | Basidiomycota |
| *Amanita rubescens* Pers.* | Amanitaceae | Agaricales | Basidiomycota |
| *Amanita* sp. 2 | Amanitaceae | Agaricales | Basidiomycota |
| *Amanita* sp. | Amanitaceae | Agaricales | Basidiomycota |
| *Aureoboletus projectellus* (Murrill) Halling * | Boletaceae | Boletales | Basidiomycota |
| *Auriscalpium vulgare* Gray | Auriscalpiaceae | Russulales | Basidiomycota |
| *Austroboletus gracilis* (Peck) Wolfe* | Boletaceae | Boletales | Basidiomycota |
| *Baeospora myosura* (Fr.) Singer | Marasmiaceae | Agaricales | Basidiomycota |
| *Boletus* G. *edulis* | Boletaceae | Boletales | Basidiomycota |
| *Byssomerulius incarnatus* (Schwein.) Gilb.* | Phanerochaetaceae | Polyporales | Basidiomycota |
| *Cantharellus cibarius* Fr.* | Cantharellaceae | Cantharellales | Basidiomycota |
| Cantharellus G. *cibarius s*p. 1 | Cantharellaceae | Cantharellales | Basidiomycota |
| Cantharellus G. *cibarius* sp. 2 | Cantharellaceae | Cantharellales | Basidiomycota |
| *Catathelasma* sp. | Tricholomataceae | Agaricales | Basidiomycota |
| *Chalciporus* sp. | Boletaceae | Boletales | Basidiomycota |
| *Chroogomphus* sp. | Gomphidiaceae | Boletales | Basidiomycota |
| *Climacocystis borealis* (Fr.) Kotl. & Pouzar | Fomitopsidaceae | Polyporales | Basidiomycota |
| *Clitocybe gibba* (Pers.) P. Kumm* | Tricholomataceae | Agaricales | Basidiomycota |
| *Clitocybula* sp. | Marasmiaceae | Agaricales | Basidiomycota |
| *Coltricia cinnamomea* (Jacq.) Murrill | Hymenochaetaceae | Hymenochaetales | Basidiomycota |
| *Cortinarius anomalus* (Fr.) Fr. | Cortinariaceae | Agaricales | Basidiomycota |
| *Cortinarius* sp. 1 | Cortinariaceae | Agaricales | Basidiomycota |
| *Cortinarius* sp. 2 | Cortinariaceae | Agaricales | Basidiomycota |
| *Cortinarius* sp. 3 | Cortinariaceae | Agaricales | Basidiomycota |
| *Cortinarius* sp. 4 | Cortinariaceae | Agaricales | Basidiomycota |
| *Cortinarius* sp. 5 | Cortinariaceae | Agaricales | Basidiomycota |
| *Cortinarius* sp. 6 | Cortinariaceae | Agaricales | Basidiomycota |
| *Cortinarius* sp. 7 | Cortinariaceae | Agaricales | Basidiomycota |
| *Cortinarius* sp. 8 | Cortinariaceae | Agaricales | Basidiomycota |
| *Cortinarius* sp. 9 | Cortinariaceae | Agaricales | Basidiomycota |
| *Craterellus cornucopioides* (L.) Pers.* | Cortinariaceae | Agaricales | Basidiomycota |
| *Craterellus tubaeformis* (Fr.) Quél.* | Cantharellaceae | Cantharellales | Basidiomycota |
| *Crepidotus* sp. | Inocybaceae | Agaricales | Basidiomycota |
| *Crepidotus* sp. | Inocybaceae | Agaricales | Basidiomycota |
| *Cystolepiota sp.* | Agaricaceae | Agaricales | Basidiomycota |
| *Dacrymyces capitatus* Schwein. | Dacrymycetaceae | Dacrymycetales | Basidiomycota |
| *Entoloma* sp. | Entolomataceae | Agaricales | Basidiomycota |
| *Galerina* sp. | Hymenogastraceae | Agaricales | Basidiomycota |
| *Gymnopilus sapineus* (Fr.) Murrill | Hymenogastraceae | Agaricales | Basidiomycota |
| *Gymnopus alkalivirens* (Singer) Halling | Omphalotaceae | Agaricales | Basidiomycota |
| *Gymnopus dryophilus* (Bull.) Murrill* | Omphalotaceae | Agaricales | Basidiomycota |
| *Gymnopus* sp. 1 | Omphalotaceae | Agaricales | Basidiomycota |
| *Hebeloma* sp. | Hymenogastraceae | Agaricales | Basidiomycota |
| *Hohenbuehelia* sp. | Pleurotaceae | Agaricales | Basidiomycota |
| *Hydnellum* sp. | Hymenogastraceae | Agaricales | Basidiomycota |
| *Hydnum repandum* L.* | Pleurotaceae | Agaricales | Basidiomycota |
| *Hygrocybe* sp. | Hygrophoraceae | Agaricales | Basidiomycota |
| *Hygrophoropsis aurantiaca* (Wulfen) Maire* | Hygrophoropsidaceae | Boletales | Basidiomycota |
| *Hygrophorus chrysodon* (Batsch) Fr.* | Hygrophoraceae | Agaricales | Basidiomycota |
| *Hygrophorus russula* (Schaeff. ex Fr.) Kauffman* | Hygrophoraceae | Agaricales | Basidiomycota |
| *Inocybe geophylla* (Bull.) P. Kumm. | Inocybaceae | Agaricales | Basidiomycota |
| *Inocybe* sp. 1 | Inocybaceae | Agaricales | Basidiomycota |
| *Inocybe* sp. 2 | Inocybaceae | Agaricales | Basidiomycota |
| *Inocybe* sp. 3 | Inocybaceae | Agaricales | Basidiomycota |
| *Inocybe* sp. 4 | Inocybaceae | Agaricales | Basidiomycota |
| *Inocybe* sp. 5 | Inocybaceae | Agaricales | Basidiomycota |
| *Inonotus* sp. | Hymenochaetaceae | Hymenochaetales | Basidiomycota |
| *Laccaria* a*methystina* Cooke* | Hydnangiaceae | Agaricales | Basidiomycota |
| *Laccaria bicolor* (Maire) P.D. Orton* | Hydnangiaceae | Agaricales | Basidiomycota |
| *Laccaria laccata* (Scop.) Cooke* | Hydnangiaceae | Agaricales | Basidiomycota |
| *Lactarius* sp. 1 | Russulaceae | Russulales | Basidiomycota |
| *Lactarius* sp. 2 | Russulaceae | Russulales | Basidiomycota |
| *Lactarius* sp. 3 | Russulaceae | Russulales | Basidiomycota |
| *Lactarius* sp. 4 | Russulaceae | Russulales | Basidiomycota |
| *Lentinus* sp. | Polyporaceae | Polyporales | Basidiomycota |
| *Lycoperdon perlatum* Pers.* | Agaricaceae | Agaricales | Basidiomycota |
| *Lepiota* sp. | Agaricaceae | Agaricales | Basidiomycota |
| *Leucoagaricus* sp. | Agaricaceae | Agaricales | Basidiomycota |
| *Marasmius* sp. 2 | Marasmiaceae | Agaricales | Basidiomycota |
| *Marasmius* sp. | Marasmiaceae | Agaricales | Basidiomycota |
| Morphospecies 10 |  |  | Basidiomycota |
| Morphospecies 11 |  |  | Basidiomycota |
| Morphospecies 12 |  |  | Basidiomycota |
| Morphospecies 13 |  |  | Basidiomycota |
| Morphospecies 14 |  |  | Basidiomycota |
| Morphospecies 3 |  |  | Basidiomycota |
| Morphospecies 4 |  |  | Basidiomycota |
| Morphospecies 5 |  |  | Basidiomycota |
| Morphospecies 6 |  |  | Basidiomycota |
| Morphospecies 7 |  |  | Basidiomycota |
| Morphospecies 8 |  |  | Basidiomycota |
| Morphospecies 9 |  |  | Basidiomycota |
| *Mycena* G. *epipterygia* | Mycenaceae | Agaricales | Basidiomycota |
| *Mycena* G. *pura* | Mycenaceae | Agaricales | Basidiomycota |
| *Mycena* sp. 1 | Mycenaceae | Agaricales | Basidiomycota |
| *Mycena* sp. 2 | Mycenaceae | Agaricales | Basidiomycota |
| *Mycena* sp. 3 | Mycenaceae | Agaricales | Basidiomycota |
| *Mycena* sp. 4 | Mycenaceae | Agaricales | Basidiomycota |
| *Mycena* sp. 5 | Mycenaceae | Agaricales | Basidiomycota |
| *Mycetinis* sp. | Omphalotaceae | Agaricales | Basidiomycota |
| *Osmoporus mexicanus* (Mont.) Ryvarden | Gloeophyllaceae | Gloeophyllales | Basidiomycota |
| *Phaeolus schweinitzii*  (Fr.) Pat. | Fomitopsidaceae | Polyporales | Basidiomycota |
| *Phaeolus* sp. | Fomitopsidaceae | Polyporales | Basidiomycota |
| *Phellodon niger* (Fr.) P. Karst.* | Bankeraceae | Thelephorales | Basidiomycota |
| *Phellodon* sp. 1 | Bankeraceae | Thelephorales | Basidiomycota |
| *Phellodon* sp. 2 | Bankeraceae | Thelephorales | Basidiomycota |
| *Pholiota* sp. | Strophariaceae | Agaricales | Basidiomycota |
| *Pluteus chrysophlebius* (Berk. & M.A. Curtis) Sacc. | Pluteaceae | Agaricales | Basidiomycota |
| polyporales Gäum. |  | Polyporales | Basidiomycota |
| *Ramaria* sp. 1 | Gomphaceae | Gomphales | Basidiomycota |
| *Ramaria* sp. 2 | Gomphaceae | Gomphales | Basidiomycota |
| *Ramaria stricta* (Pers.) Quél.* | Gomphaceae | Gomphales | Basidiomycota |
| *Rhodocollybia butyracea* (Bull.) Lennox* | Omphalotaceae | Agaricales | Basidiomycota |
| *Russula brevipes* Peck* | Russulaceae | Russulales | Basidiomycota |
| *Russula* G. *emetica* Peck | Russulaceae | Russulales | Basidiomycota |
| *Russula* sp. 1 | Russulaceae | Russulales | Basidiomycota |
| *Russula* sp. 2 | Russulaceae | Russulales | Basidiomycota |
| *Russula* sp. 3 | Russulaceae | Russulales | Basidiomycota |
| *Stereum* aff. o*strea* (Blume & T. Nees) Fr. | Stereaceae | Russulales | Basidiomycota |
| *Stereum* sp. | Stereaceae | Russulales | Basidiomycota |
| *Suillus* sp. 1 | Suillaceae | Boletales | Basidiomycota |
| *Suillus* sp. 2 | Suillaceae | Boletales | Basidiomycota |
| *Trametes* aff. *Villosa* (Sw.) Kreisel | Polyporaceae | Polyporales | Basidiomycota |
| *Trichaptum abietinum* (Pers.) Ryvarden | Polyporaceae | Hymenochaetales | Basidiomycota |
| *Tricholoma equestre* (L.) P. Kumm | Tricholomataceae | Agaricales | Basidiomycota |
| *Tricholoma* sp. 1 | Tricholomataceae | Agaricales | Basidiomycota |
| *Tricholoma* sp. 2 | Tricholomataceae | Agaricales | Basidiomycota |
| *Tricholoma* sp. 3 | Tricholomataceae | Agaricales | Basidiomycota |
| *Tricholoma* sp. 4 | Tricholomataceae | Agaricales | Basidiomycota |
| *Tricholoma* sp. 5 | Tricholomataceae | Agaricales | Basidiomycota |
| *Tricholoma* sp. 6 | Tricholomataceae | Agaricales | Basidiomycota |
| *Tricholoma* sp. 7 | Tricholomataceae | Agaricales | Basidiomycota |
| *Xeromphalina campanella* (Batsch) Kühner & Maire | Mycenaceae | Agaricales | Basidiomycota |
| *Xerula* sp. | Physalacriaceae | Agaricales | Basidiomycota |
